# The Diagnosis and Treatment of Diseases of the Rectum

**Published:** 1888-12

**Authors:** 


*The Diagnosis and Treatment of Diseases of the Rectum.* By WM. ALLINGHAM, F.R.C.S. Fifth Edition. Edited by HERBERT WM. ALLINGHAM, F.R.C.S. Pp. 366. London: J. & A. CHURCHILL. 1888.

The value of this excellent and well-known work of Mr. Allingham has been much enhanced by revisions and additions made by his son. We would refer to the chapters on Excision of the Rectum and Inguinal Colotomy as being specially good. Mr. Allingham, junior, has the credit of having introduced a method of inguinal colotomy which promises to take a permanent place among surgical procedures: this operation is clearly and modestly described. This is not the place in which to compare the relative values of inguinal and lumbar colotomy; but if lumbar colotomy should have to give way to inguinal, as seems not unlikely, the influence of the younger Allingham's operation in determining the choice must be not inconsiderable.

